# Initial IL-10 production dominates the therapy of mesenchymal stem cell scaffold in spinal cord injury

**DOI:** 10.7150/thno.87843

**Published:** 2024-01-01

**Authors:** Lijun Yang, Jian Cao, Yiwen Du, Xunqi Zhang, Wenxiang Hong, Bowen Peng, Jiahe Wu, Qinjie Weng, Jiajia Wang, Jianqing Gao

**Affiliations:** 1 Center for Drug Safety Evaluation and Research, College of Pharmaceutical Sciences, Zhejiang University, Hangzhou 310058, China.; 2 Nanhu Brain-computer Interface Institute, Hangzhou, 311100, China.; 3 Zhejiang Province Key Laboratory of Anti-Cancer Drug Research, College of Pharmaceutical Sciences, Zhejiang University, Hangzhou 310058, China.; 4 Institute of Pharmaceutics, College of Pharmaceutical Sciences, Zhejiang University, Hangzhou 310058, China.; 5 Dr. Li Dak Sum & Yip Yio Chin Center for Stem Cell and Regenerative Medicine, Zhejiang University, Hangzhou 310058, China.; 6 Department of Pharmacy, The Second Affiliated Hospital, Zhejiang University School of Medicine, Hangzhou 310009, China.; 7 National Key Laboratory of Advanced Drug Delivery and Release Systems, College of Pharmaceutical Sciences, Zhejiang University, Hangzhou 310058, China.

**Keywords:** mesenchymal stem cells, IL-10, spinal cord injury, cell migration, cytokines secretion

## Abstract

**Rationale:** Spinal cord injury (SCI) is an acute damage to the central nervous system that results in severe morbidity and permanent disability. Locally implanted scaffold systems with immobilized mesenchymal stem cells (MSCs) have been widely proven to promote locomotor function recovery in SCI rats; however, the underlying mechanism remains elusive.

**Methods and Results:** In this study, we constructed a hyaluronic acid scaffold system (HA-MSC) to accelerate the adhesive growth of human MSCs and prolong their survival time in SCI rat lesions. MSCs regulate local immune responses by upregulating the expression of anti-inflammatory cytokines. Interestingly, the dramatically increased, but transient expression of interleukin 10 (IL-10) is found to be secreted by MSCs in the first week. Blocking the function of the initially produced IL-10 by the antibody completely abolished the neurological and behavioral recovery of SCI rats, indicating a core role of IL-10 in SCI therapy with HA-MSC implantation. Transcriptome analyses indicated that IL-10 selectively promotes the migration and cytokine secretion-associated programs of MSCs, which in turn helps MSCs exert their anti-inflammatory therapeutic effects.

**Conclusion:** Our findings highlight a novel role of IL-10 in regulating MSC migration and cytokine secretion-associated programs, and determine the vital role of IL-10 in the domination of MSC treatment for spinal cord repair.

## Introduction

Mesenchymal stem cells (MSCs) are mesodermal lineage multipotent progenitors with self-renewal and multipotency capacities that have been widely used in clinical and pre-clinical studies of neurodegenerative and neurotraumatic diseases 1-4. MSCs can migrate to injured neurons, inhibit mitochondrial oxidative stress, and differentiate into neuron-like cells to reduce neuronal apoptosis and promote neural regeneration 5-7. In addition, some trophic factors released by MSCs, such as brain-derived neurotrophic factor (BDNF) and nerve growth factor (NGF), have protective effects on the neurological, immune, and metabolic systems 8, 9. Moreover, an increasing number of studies have shown that MSCs exert an anti-inflammatory effect by regulating immune cells, such as macrophages and T cells 10, providing new options for MSCs to inflammatory disease treatment.

Spinal cord injury (SCI) is an acute damage that is usually caused by an external physical impact, such as a motor vehicle injury or fall, dramatically impairing bio-message transmission and leading to devastating consequences like paralysis and even death 11. During SCI, the primary insult causes neural cell death and triggers a secondary injury cascade, including inflammation, resulting in further deterioration. Severe inflammation is the main obstacle to overcome in the acute phase of SCI 12. In recent decades, MSCs have shown great promise for SCI treatment owing to their low immunogenicity, wide range of sources, and multiple bioactivities, including anti-inflammatory, immunomodulatory, pro-angiogenic, trophic, and paracrine effects 13-16. However, the molecular mechanisms underlying MSC therapy for SCI remain unclear.

Notably, the harsh local microenvironment following SCI, such as increased immune responses and flushing of blood and cerebrospinal fluid, severely endangers the accumulation and survival of transplanted MSCs, thereby compromising their therapeutic effects 17. Hence, biomaterials used to encapsulate MSCs has emerged as a promising strategy for providing a structural substrate for MSC survival and growth 18. According to our previous work, an adhesive peptide (PPFLMLLKGSTR), derived from the laminin-5 α3 chain and had a natural affinity to the MSC-expressed integrin, was modified into a hyaluronic acid (HA) scaffold for human MSC encapsulation and adhesive growth. As expected, HA scaffold-immobilized human MSCs (HA-MSC) showed better locomotor function recovery and spinal cord regeneration than did MSCs implanted into SCI rats 17, 19. In fact, we did not open the black box of HA-MSC and uncovered the key factors in SCI therapy using HA-MSC.

In the present study, HA-MSC were implanted into rats with SCI to determine the underlying mechanism of MSC-mediated SCI repair. Higher retention of MSCs on the HA scaffold was observed in SCI rats, contributing to the repression of inflammation. Interestingly, the transient but copious secretion of human interleukin 10 (IL-10) by MSCs promotes injured spinal cord repair. In addition to its anti-inflammatory role, IL-10 can increase the cytokine secretion associated pathways and migration of MSCs, which may also contribute to the spinal cord restoration by MSCs. In summary, our findings revealed the dominant role of IL-10 in MSC treatment for spinal cord repair.

## Materials and methods

### Materials

Hyaluronic acid was purchased from Bloom Biotechnology (Jinan, China). The adhesive peptide (sequence: PPFLMLLKGSTR) was synthesized by BankPeptide Biological Technology (Hefei, China). Ethyl N, N-dimethylaminopropyl carbodiimide (EDC) was purchased from Merck (Shanghai, China). Adipic dihydrazide (ADH), 1-hydroxybenzotriazole (HOBt) and sodium metapriodate (NaIO_4_) were purchased from Macklin (Shanghai, China). The GFP transfection kit was obtained from GeneChem (Shanghai, China). A live/dead assay kit was purchased from Beyotime Biotechnology (Shanghai, China).

Human umbilical cord MSCs were provided by SinoCell Technology Ltd. (Ningbo, China), and the study was approved by Ningbo First Hospital (Ethics number: 2021-R108). The culture medium consisted of MEM-II, 10% fetal bovine serum, human FGF (20 ng/mL), and human EGF (20 ng/mL). GFP-expressing MSCs were constructed by lentiviral transfection using a GFP-transfection Kit (GeneChem, Shanghai, China).

Female SD rats weighing approximately 230 g were purchased from SLAC Laboratory Animal Co., Ltd. (Shanghai, China). All animal experiments were conducted under the guidance of the Animal Ethics Committee of Zhejiang University and approved by the Institutional Animal Care and Use Committee (IACUC) of Zhejiang University (IACUC number: IACUC-23-446).

### Preparation and characterization of peptide-modified scaffold

ADH and NaIO_4_ were used to introduce an aldehyde group (-CHO) and an amino group (-NH_2_) onto the HA chain to obtain HA-ADH and HA-CHO, as described previously 17. For peptide modification, adhesive peptide (5 mg) was dissolved in DMSO (500 μL) and then added into HA-CHO solution (10 mL, 10 mg/mL) to substitute partial -CHO of HA-CHO. After 2 h, the mixture was dialyzed against a 3 KDa MWCO in DI water for purification. The product was obtained by freeze drying. The chemical structures of the reactants and products were confirmed by nuclear magnetic resonance (NMR, AVANCE 500 M, Bruker) and Fourier transform infrared spectroscopy (FTIR, NICOLET iS50FT-IR, Thermo Scientific).

Thirty microliters of HA-ADH (12.5 mg/mL) and an equal volume of the HA peptide (15 mg/mL) were mixed and cast into a 384-well plate to form a cylindrical scaffold. The scaffolds obtained were freeze-dried for storage. The inner structure of the scaffold was examined using scanning electron microscopy (SEM, Nova Nano 450, Thermo FEI) and micro-CT (Skyscan1272, Bruker Technology Co., Ltd.). The reconstructed 3D image of the scaffold was obtained using the Avizo software (Thermo Scientific) to calculate the volume fraction, pore area, and pore volume. The rheological properties of the scaffolds were measured using rheometer (Mars40, Thermo Fisher Scientific). To detect degradation, the HA scaffold was immersed in PBS at different pH values (7, 6.6, 5.8, and 3). The *in vivo* degradation was also detected in SCI rats.

For MSC encapsulation, the freeze-dried scaffold was immersed, swollen in PBS, and sterilized using a 75% ethanol solution. The swelling ratio or water content of the scaffolds was determined by measuring the changes in weight. The swelled scaffold was dehydrated using sterile filter paper and then cell suspension (30 μL) containing 10^5^ MSCs was injected into the scaffold and cultured in culture medium overnights for cell adhesion. Cell survival in the scaffold was detected at 12 h and 5 d using a live/dead assay kit.

### MSC distribution after implanting into the spinal cord

To investigate the* in vivo* fate of MSCs after implantation into the damaged spinal cord, the MSCs were stained with the membrane dye DiR. The SCI model was established in SD rats. Briefly, the spinal cord at T10 was exposed via laminectomy and fully transected to form a lesion gap (3 mm). DiR-stained MSCs (cell number: 10^5^) were locally injected (MSC group) or implanted with scaffolds (HA-MSC group) into lesions, followed by complete hemostasis. The rats were sacrificed and perfused with PBS and paraformaldehyde (PFA) at 12 and 24 h to collect the heart, liver, spleen, lung, kidney, and spinal cord. *In vivo* MSC distribution was imaged using *in vivo* imaging system (U-CT-XUHR; Milabs, Netherlands).

### *In vivo* MSC efficacy after IL-10 blocking

Fully transfected SCI models were constructed as previously described. MSCs were transplanted with (HA-MSC group) or without (MSC group) the HA scaffold into the lesions. A blank HA scaffold was also implanted as a control (HA-null group). To investigate the role of IL-10 in MSC therapeutics, an IL-10 antibody (30 μL, 2 mg/mL) was locally injected into the scaffold (HA-MSC+hAnti-IL-10) and intravenously injected on day 7. The body weights of the rats in each group were measured, and Basso, Beattie, and Bresnahan (BBB) scores were evaluated by recording free walking in an open field weekly. On day 35, the rats were placed on a ladder to test creep from one end to the other. After the ladder test, the rats were sacrificed and perfused with PBS and paraformaldehyde (PFA). The spinal cord was collected and sectioned for Masson and immunofluorescence staining.

### RNA extraction and Quantitative real-time PCR

Spinal cords were collected on day 1, 4, and 7 from rats implanted with the MSC scaffolds and on day 7 from rats implanted with anti-IL-10 MSC scaffolds. RNA was isolated using TRIzol (Invitrogen), and the TransScript kit (TransGen Biotech) was used to reverse transcribe the RNA into cDNA. And the qRT-PCR analysis was performed with SYBR Green (Bio-Rad), DEPC water and primers in a total 20 μL mixture (ABI Fast 7500). All data were normalized to *GAPDH*.

### Western blotting

Spinal cords were collected on day 1 and 4 from rats implanted with MSC scaffolds and cells treated with recombined human IL-10 (rhIL-10) for 8 h. Total protein was quantification with BCA after lysis in lysis buffer. Proteins were separated by 8-12% SDS-PAGE and transferred to a polyvinylidene fluoride (PVDF) membrane. 5% fat-free milk was used for blocking, and T-PBS was used for washing three times. Then, the membranes were cut with molecular weight and incubated with primary antibody. The following primary antibodies were used: Cell Signaling Technology: anti-p-NF-κB (#3033), anti-JAK2 (#3230), anti-p-JAK2 (Tyr1007/1008) (#3771), anti-STAT3 (#9139,), anti-p-STAT3 (Y705) (#9145); Huabio: anti-NF-κB (ET1603-12,), anti-CCR7 (ET1602-22); DiagBio Technology.: anti-GAPDH (db106, 1:5000); Santa Cruz biotechnology: anti-Vimentin (sc-6260). Horseradish peroxidase (HRP)-conjugated IgG (MULTI Sciences) was used to bind the primary antibody for 90 mins at room temperature, and enhanced chemiluminescence (ECL, PerkinElmer) was added to the strip to detect the target protein; images were captured with a digital imager (azure, c280). GAPDH was used as a loading control.

### Transwell migration assay

The inserts were placed in 24-well plates and MSCs (2 × 10^4^ cells) were seeded onto the inserts, with rhIL-10 or not. After 8 h, the cell culture media were removed from both the insert and 24-well plate, the cells were fixed with 4% PFA for 30 min and stained with crystal violet. After removing the cells above the membrane with a cotton swab, images were captured using a microscope (Lecia, DM2500) and the migrated cells were counted.

### Immunofluorescence staining

Permeabilization buffer (PBS containing 0.5% Triton X-100) was added to the spinal cord sections or MSCs for 10 min, followed by blocking buffer (PBS containing 0.5% Triton X-100 and sheep serum) for 1 h, washing the sections with PBS for 3 times and adding primary antibody to incubate at 4 °C overnight. The following primary antibodies were used: Santa Cruz: anti-GFP antibody (E022200), Abcam: anti-human Nuclear Antigen antibody (ab191181); Beyotime Biotechnology: Anti-Tracker Green-488 (C2201S); Cell Signaling Technology: Anti-NF antibody (2836). After washing three times with PBS, the sections were incubated with a secondary antibody at room temperature for 90 min; Alexa Fluor 488-coupled secondary antibodies (A-21202,) were purchased from Life Technologies, the nuclei were counterstained with DAPI (Beijing Solarbio Science & Technology Co., Ltd.), and images were captured using a confocal microscope (Leica, TCS SP8).

### RNA-seq and data analysis

The SMARTER mRNA-Seq Library Prep Kit was used to prepare RNA-seq libraries for MSCs treated with rhIL-10 or not. All RNA-seq data were aligned to hg38 using TopHat with the default settings (http://tophat.cbcb.umd.edu/). All high-throughput data were deposited in the NCBI Gene Expression Omnibus (GEO) database under the accession number GEO: GSE240755.

### Statistical analysis

The data were presented as mean ± SD and analyzed using GraphPad Prism software with Student's t-test or one-way ANOVA methods. Statistical significance was set at * *P* < 0.05; ** *P* < 0.01; *** *P* < 0.001.

## Results

### HA Scaffold supports adhesive growth of MSCs

**Figure 1:**
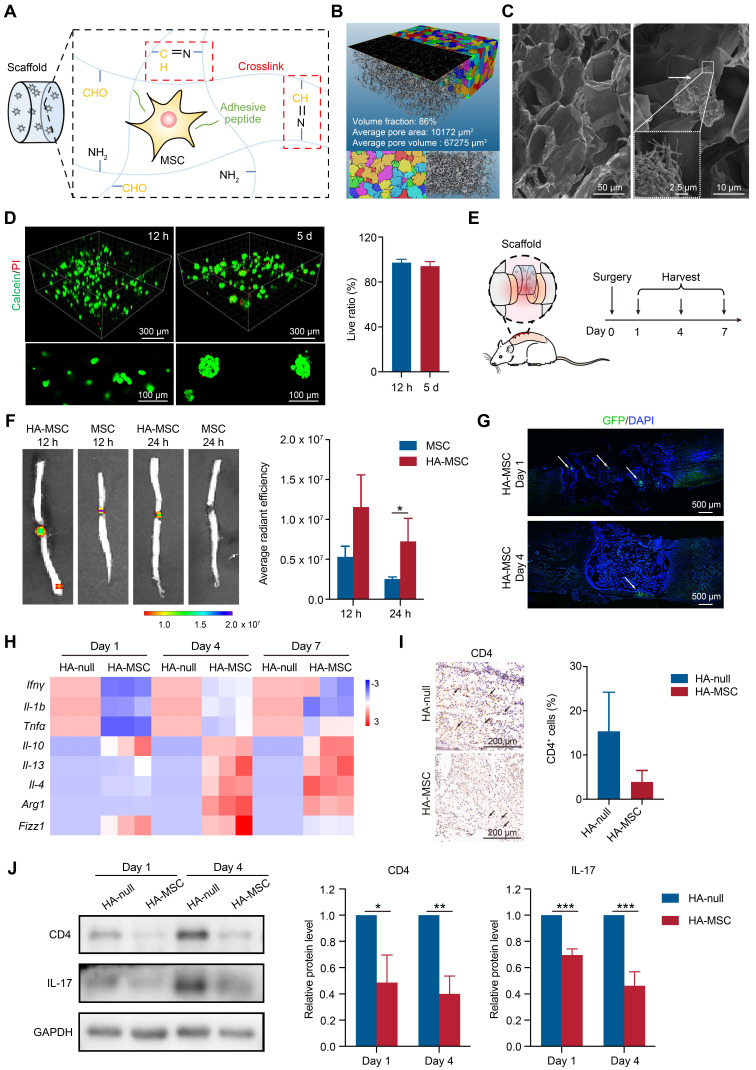
** HA-MSC scaffold implantation inhibits the inflammation in SCI rats.** (A) Diagram showing the preparation of HA scaffold and MSC encapsulation. (B) The 3D image and pore distribution of HA scaffold based on microCT scanning. (C) The microstructure of HA scaffold (magnification time: 400) and MSC adhesion on scaffold (magnification time: 2000) scanned by SEM. Arrows indicate MSCs. (D) Live (green)/dead (red) assay for MSCs cultured in HA scaffold for 12 h and 5 d. (E) Diagram showing surgery and harvest schedule. (F) DiR-labelled MSCs were injected or implanted with HA scaffold and the cell distribution was observed by *in vivo* imaging at 12 and 24 h (n = 3 animals per group). (G) Immunostaining of GFP-MSCs (green) in spinal cord when implanted with HA scaffold on day 1 and 4. Arrows indicated MSCs (n = 3 animals per group). (H) Relative mRNA expression of pro-inflammatory factors, anti-inflammatory factors and M2 macrophage markers in rats treated with scaffold and HA-MSC on day 1, 4 and 7 (n = 3 animals per group). (I) Immunohistochemistry for CD4 in spinal cord for HA-null and HA-MSC groups (n = 3 animals per group). (J) Western blotting analysis and quantification for the expression of CD4 and IL-17 in spinal cord for HA-null and HA-MSC groups (n = 3 animals per group).

For MSC encapsulation and adhesive growth, HA scaffolds were constructed based on the rapid crosslinking of -NH_2_ on HA-adipic dihydrazide and -CHO on the HA-peptide (Figure 1A). The scaffolds were successfully synthesized and confirmed using nuclear magnetic resonance and Fourier transform infrared spectroscopy (Figure S1A-B). The scaffold showed an elastic modulus (G') similar to that of the spinal cord (0.1~16 kPa) and rapid swelling behavior within 30 min in PBS (Figure S1C-D). Because of the pH-responsive characteristics of the Schiff base in the scaffold, we performed *in vitro* and *in vivo* scaffold degradation assays. The results showed that the scaffold quickly degraded within 5 days when immersed in a PBS solution at pH 3, while maintaining slow degradation in mildly acidic or neutral pH conditions (Figure S1E). Consistently, the scaffold in the SCI rats gradually degraded and was replaced with newborn tissue within 4 weeks (Figure S1F). The scaffold was scanned using micro-CT, and the reconstructed image showed the porous structure of the HA scaffold with approximately 86% volume fraction. The pores are labeled with different colors to represent different sizes. The porous scaffold, which has an average pore area of 10172 μm^2^ and pore volume of 67275 μm^3^ (Figure 1B), was deemed as a suitable live space for MSCs 19. As expected, the SEM images also confirmed the porous inner structures of the HA scaffold, and the adhesive growth of MSCs was detected in the HA scaffold owing to the affinity between the peptide and MSCs (Figure 1C). In addition, the live/dead assay results showed a high survival rate of MSCs after culturing for 12 h and 5 d in the above HA scaffold, indicating the excellent biocompatibility of the HA scaffold (Figure 1D). Interestingly, we also observed that MSCs gradually formed single cells into sphere-like aggregates after culturing in the scaffold for 5 d (Figure 1D). Thus, we concluded that the HA scaffold was suitable for the adhesive growth of MSCs.

### HA Scaffold improves MSC survival to inhibit neuroinflammation after SCI

To directly visualize MSCs *in vivo*, MSCs were stained with DiR for *in vivo* imaging or transfected to stably express GFP for immunofluorescence. The rat spinal cords were transected, DiR-MSCs or GFP-MSCs with or without HA scaffolds were implanted into the lesion site, and the spinal cords were harvested for analysis on day 1, 4, and 7 (Figure 1E). During the first day post-injury, MSC signals were dramatically decreased due to the flushing of blood and cerebrospinal fluid and increased immune response, as shown by *in vivo* imaging (Figure 1F). In contrast, HA-MSC remained at a relatively high level of signals at the lesion site (Figure 1F-G), indicating that the HA scaffolds exhibited excellent MSC encapsulation and preserved MSCs in SCI rats. These surviving MSCs then began to migrate out of the scaffold and to the adjacent injured spinal cords in the following days (Figure 1G), where they were attracted by the cytokines secreted in the microenvironment 19.

MSCs at the injured site inhibit neuroinflammation and accelerate nerve regeneration 20. As expected, elevated superoxide dismutase (SOD) activity and decreased malondialdehyde (MDA) levels were observed in HA-MSC-implanted spinal cords from day 1 to 7 (Figure S2), indicating the protection of MSCs against the reactive oxygen species (ROS) microenvironment.

Consistently, the gene expressions of pro-inflammatory cytokines, including *Ifnγ*, *Il-1b*, and *Tnfα*, were decreased, while the gene levels of anti-inflammatory cytokines, including* Il-10*, *Il-13*, and *Il-4*, were increased after HA-MSC implantation (Figure 1H). MSCs regulate macrophage polarization, thus promoting SCI recovery 21. We detected the RNA levels of markers for M2-polarized macrophages and found that the RNA levels of *Arg1*, *Fizz1* and *Il-4* were up-regulated in spinal cord lesions from the HA-MSC group (Figure 1H). Additionally, the number of CD4^+^ T cells and the expression of inflammatory factors such as IL-17 decreased in the HA-MSC group, as indicated by immunohistochemistry and western blotting (Figure 1I-J). In summary, HA scaffolds effectively promoted the accumulation and survival of MSCs, and HA-MSC could regulate macrophages and T cells to inhibit neuroinflammation.

### MSCs secrete transient but copious human cytokine IL-10 in SCI lesion

**Figure 2:**
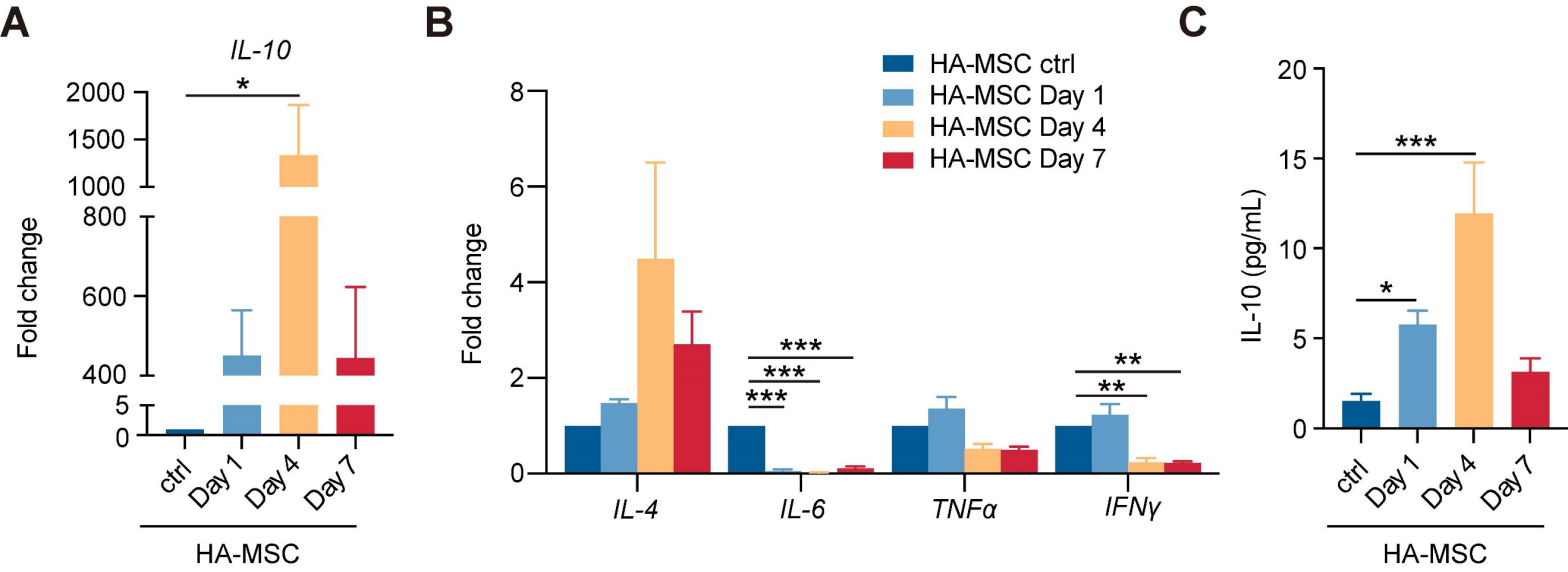
** Transient increase of hIL-10 secreted by MSCs.** (A-B) Relative mRNA expression of IL-10 (A) and inflammation-related factors (B) in rats treated with HA-MSC on day 1, 4 and 7 (n = 3 animals per group). (C) Protein level of IL-10 in MSC scaffold from rats with SCI on day 1, 4 and 7 (n = 3 animals per group).

Given the paracrine characteristics of MSC in immunoregulation 22, we next collected lesion tissues with the HA scaffolds to investigate the gene expression of human cytokines in MSC-treated SCI rats. Interestingly, the gene expression of human *IL-10* was the most significantly upregulated detected by qRT-PCR analysis, up to 1000 folds at day 4, and decreased at day 7 (Figure 2A). The gene expression of anti-inflammatory factors, such as human *IL-4*, gradually increased, and that of pro-inflammatory factors decreases over time (Figure 2B). Similar results were found in *in vitro* studies, bone-marrow-derived macrophages (BMDM) or peripheral blood mononuclear cells (PBMC) were co-cultured with MSCs. Compared with MSC group, the expression of *IFN-γ*, *IL-6*, *IL-4* and *IL-13* were altered while the expression of *IL-10* was increased in co-cultured groups (Figure S3). Using ELISA analysis, we further confirmed the increased secretion of human IL-10 from day 4 to peak, consistent with its transcriptional change (Figure 2C). Thus, we concluded that there was a transient but copious secretion of human IL-10 by MSCs in HA-MSC-treated SCI lesions.

### Human IL-10 antibody abolishes the functional recovery of spinal cord by HA-MSC scaffolds

**Figure 3:**
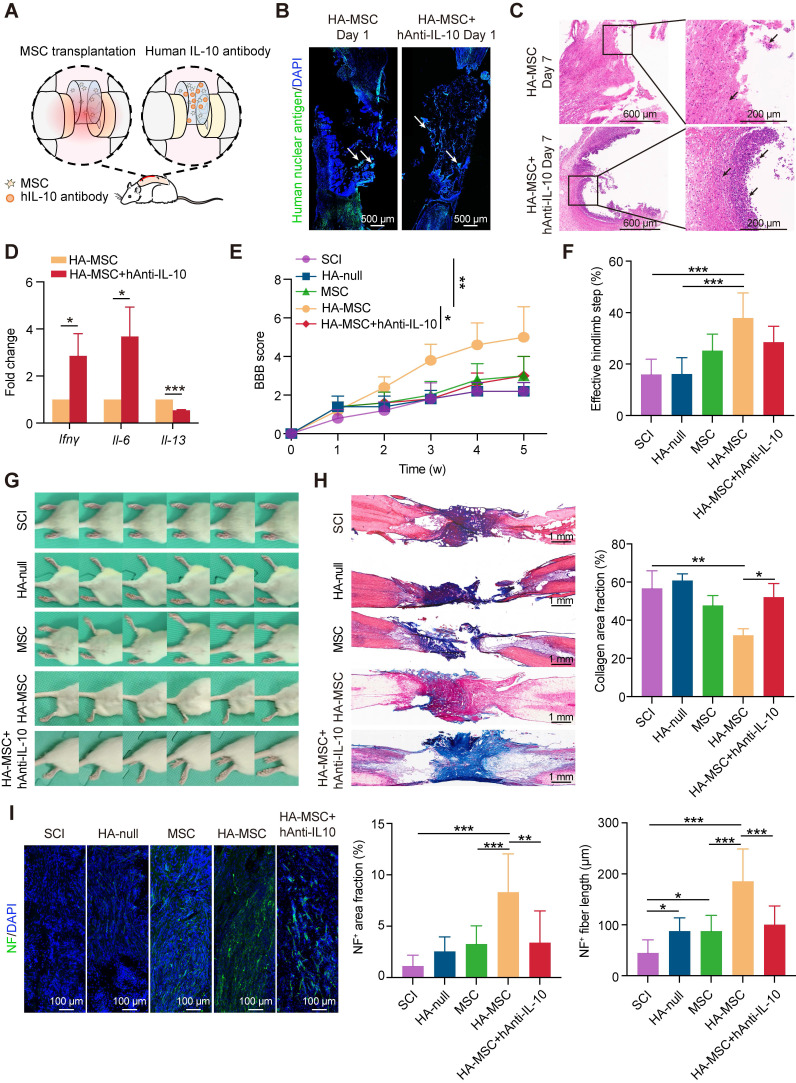
** Human IL-10 production by MSCs contributes to spinal cord recovery.** (A) Schematic diagram of rats treated with HA-MSC or HA-MSC+hAnti-IL-10. (B) Immunostaining of MSC (green) in spinal cord when implanted with MSC or anti-IL-10 treated MSC scaffold on day 1. Arrows indicated MSCs. (C) H&E staining of spinal cords near the scaffold in HA-MSC- and HA-MSC + hAnti-IL-10-treated SCI rats on day 7. (D) Relative mRNA expression of pro-inflammatory and anti-inflammatory factors in rats treated with vehicle and hAnti-IL-10 on day 7 (n = 3 independent experiment). (E-G) BBB scores (E), effective hindlimb step ratio (F) and typical records of animal walking gait on day 35 showing hindlimb walking patterns (G) of rats in each group (n = 5 animals per group). (H) Masson staining of the spinal cord and collagen area fraction in each group (n = 5 animals per group). (I) Nerve regeneration and NF^+^ area fraction, NF^+^ fiber length in the lesion of spinal cord (n = 5 animals per group).

To investigate whether transient human IL-10 influences MSC-mediated SCI recovery, we injected a human IL-10 antibody (hAnti-IL-10) into HA-MSC to block IL-10 and implanted them into SCI rats (Figure 3A). Immunostaining for the human nuclear antigen (indicating the MSC nucleus) showed that the distribution of MSCs in the scaffold on day 1 did not change after hAnti-IL-10 application, indicating that IL-10 did not affect MSC residency (Figure 3B). In addition, cell viability and western blotting analyses demonstrated that IL-10 did not affect MSC growth or survival (Figure S4). However, H&E staining revealed increased immune cell infiltration in hAnti-IL-10 treated lesion site (Figure 3C). Besides, the expression of pro-inflammation genes *Il-6* and *Ifnγ* was elevated, while the level of anti-inflammation gene *Il-13* was decreased at day 7 after hAnti-IL-10 treatment (Figure 3D), suggesting that blocking human IL-10 in MSCs suppresses the anti-inflammatory effects of MSCs.

We speculated that neutralizing MSC-secreted IL-10 would compromise the functional recovery of HA-MSC during the chronic phase. After 5 weeks, motor function recovery in the SCI rats was evaluated using BBB scoring and the ladder walking test 23.

Results indicated that SCI rats treated with HA-MSC obtained rapid functional recovery and retrieved a BBB score of 5.00 ± 1.58 on day 35, much higher than that of the SCI group (2.00 ± 0.71), HA-null group (2.20 ± 0.45), and MSC group (3.00 ± 1.00) (Figure 3E, Video S1). However, hAnti-IL-10 treatment almost completely abolished the protective effect of HA-MSC, with a BBB score of 3.00 ± 1.00. Similar results were consistently observed in the statistics of effective hind limb steps in the ladder walking test. Rats in HA-MSC group obtained the highest effective hindlimb step ratio (37.91 ± 9.79%) while only 28.48 ± 6.21% was found in HA-MSC+hAnti-IL-10 groups, indicating the coordinated movement of rats was blocked by hAnti-IL-10 (Figure 3F). Rats in the SCI and HA-null treatment groups exhibited sweeping of the hind limb without joint movement on day 35. HA-MSC treatment resulted in distinct movement of the ankle joints and frequent weight-supported plantar steps of the hind limb, whereas SCI rats that received HA-MSC with hAnti-IL-10 remained drag movement (Figure 3G). These results suggested that human IL-10 blocking suppresses the therapeutic effects of HA-MSC in SCI rats.

Histological nerve restoration was evaluated based on stromal scarring and neurofilament (NF)-positive axons 24. Masson's trichrome staining combined with stromal scarring analysis of the lesion site on day 35 showed that the significant alleviation of scar formation induced by HA-MSC was completely abolished by hAnti-IL-10 (Figure 3H). Immunostaining results indicated that the increased NF^+^ neuron area and fiber length induced by HA-MSC were significantly reduced after hAnti-IL-10 application (Figure 3I). Taken together, these data suggest that the initiation of human IL-10 production by MSCs plays a key role in HA-MSC therapy during spinal cord regeneration.

### IL-10 enhances the cytokine secretion associated pathways and migration of MSC

**Figure 4:**
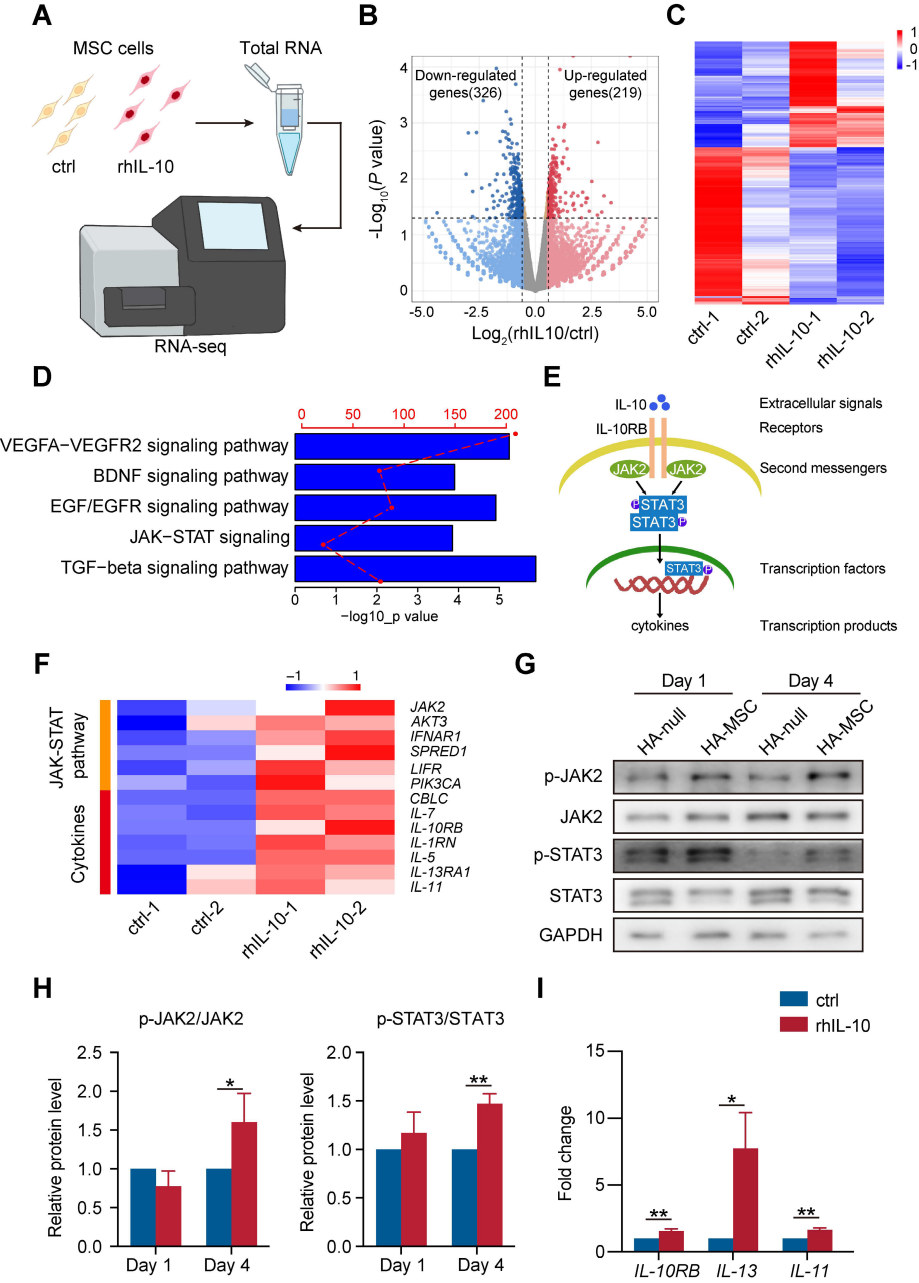
** IL-10 promotes cytokine secretion-related pathways in MSCs.** (A) Schematic diagram of the workflow for obtaining MSCs treated with rhIL-10 or not for RNA-seq. (B) Volcano plot of genes differentially regulated in IL-10-treated MSCs (fold change >1.5; *P* < 0.05). (n = 2 independent experiments). (C) Heatmap of differentially expressed genes in IL-10-treated MSCs from two samples per group. (D) Enrichment analyses of secretion pathways that up-regulated in IL-10-treated MSCs. (E) Schematic diagram of JAK-STAT pathway. (F) Heatmap analyses of up-regulated genes associated with JAK-STAT pathway and cytokines in IL-10-treated MSCs. (G-H) Western blotting analysis (G) and quantification (H) for the expression of JAK-STAT pathway proteins in the anterior spinal cord from SCI rat (n = 3 independent experiments). (I) Relative mRNA expression of IL-10RB and cytokines in MSCs treated with vehicle and rhIL-10 for 8 h (n = 3 independent experiments).

IL-10 is an anti-inflammatory cytokine that participates in the inhibition of immune responses after spinal cord transection 25. However, the role of IL-10 in the MSC function remains elusive. Therefore, we incubated MSCs with rhIL-10 for 8 h and collected for transcriptome profiling (Figure 4A). rhIL-10 treatment resulted in significant gene expression changes (fold change > 1.5, *P* < 0.05) in MSCs compared with control MSCs (Figure 4B-C). Moreover, enrichment analyses revealed that genes up-regulated in rhIL-10 treated MSCs were enriched in cytokine secretion associated pathways, such as JAK-STAT signaling and TGF-beta signaling pathway (Figure 4D).

JAK2/STAT3 is a classic pathway associated with inflammation (Figure 4E); once IL-10 binds to its receptor (IL-10RB), this interaction leads to the phosphorylation of JAK2 (second messengers). Once JAK2 is phosphorylated, its activated kinase domain enables the phosphorylation of STAT3 (a transcription factor), thus promoting the secretion of cytokines (transcription products). Our heatmap showed that genes involved in the JAK-STAT signaling pathway and cytokines, such as *IL-7*, *IL10RB*, and* IL-5*, were up-regulated in rhIL-10 treated MSCs in contrast to the control group (Figure 4F). Consistently, a subsequent western blotting analysis of the injured spinal cord showed that phosphorylated JAK2 (p-JAK2) and phosphorylated STAT3 (p-STAT3) were increased in the HA-MSC group, indicating the activation of JAK2-STAT3 signaling *in vivo* (Figure 4G-H). In addition, the RNA levels of the IL-10 receptor *IL-10RB* and the inflammatory cytokines *IL-13* and *IL-11* were raised in rhIL-10 treated MSCs (Figure 4I). Thus, we concluded that initiating IL-10 production by MSC enhanced the cytokine secretion-associated pathway of MSCs during SCI.

**Figure 5:**
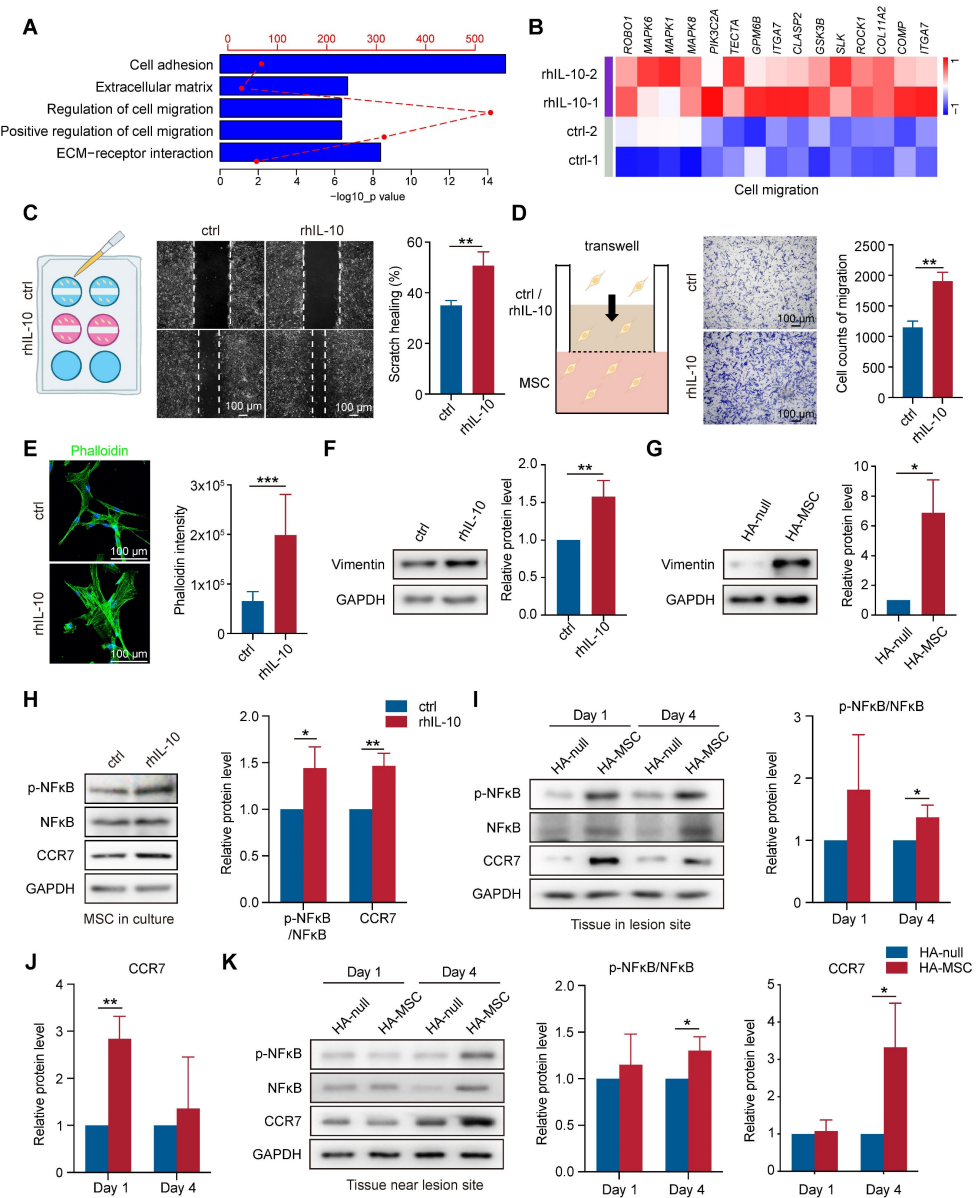
** IL-10 promotes migration of MSCs.** (A) Enrichment analyses of migration-associated pathways that up-regulated in IL-10-treated MSCs. (B) Heatmap analyses migration-associated genes up-regulated in IL-10-treated MSCs. (C) Schematic diagram, representative images and quantification of scratch healing in MSCs treated with rhIL-10 or not (n = 3 independent experiments). (D) Schematic diagram, representative images and quantification of transwell experiment in MSCs treated with rhIL-10 or not (n = 3 independent experiments). (E) Immunostaining and quantification of Phalloidin (green) for MSCs treated with rhIL-10 or not (n = 3 independent experiments). (F-G) Western blotting analysis and quantification for the expression of EMT associated protein in MSCs treated with rhIL-10 or not for 8 hours (F) or tissues from HA-null or HA-MSC treated rats for 4 days (G) (n = 3 independent experiments). (H) Western blotting analysis and quantification for the expression of migration-associated proteins in MSCs treated with rhIL-10 or not (n = 3 independent experiments). (I-K) Western blotting analysis and quantification for the expression of migration-associated proteins in the lesion site tissue (I-J) or tissue near lesion site (K) from SCI rat models (n = 3 independent experiments).

In addition to the regulation of IL-10 on cytokine secretion, we observed that gene signatures related to cell migration, cell adhesion, and extracellular matrix were enriched in rhIL-10-treated MSCs (Figure 5A). Cell adhesion and the extracellular matrix organize signaling networks direct cell migration 26, 27. The heatmap also showed upregulated expression of migration-associated genes, such as *ITGA7, and MAPK6*, in rhIL-10-treated MSCs (Figure 5B). We performed scratch and transwell assays to examine whether IL-10 directly promotes MSC migration. Notably, rhIL-10 markedly increased the scratch healing rate and the number of migrated MSCs (Figure 5C-D). Immunostaining for phalloidin showed the same results: rhIL-10 treatment enhanced fiber formation, indicating a better migration effect (Figure 5E). Epithelial-mesenchymal transition (EMT) is essential for cell migration 28, and our western blotting results showed the upregulation of Vimentin in rhIL-10 treated MSCs, as well as in lesions from the HA-MSC group (Figure 5F-G). The transcription factor nuclear factor kappa B (NF-κB) and the C-C chemokine receptor type 7 (CCR7) serve as pivotal mediators of inflammatory responses as well as cell migration 29, 30. Western blotting results showed that both phosphorylated NF-κB (p-NF-κB) and CCR7 expression were elevated not only in MSCs after rhIL-10 treatment, but also in or near the injured spinal cords (Figure 5H-K). Taken together, these results suggest that MSCs secrete the transient cytokine IL-10 to promote MSC itself to migrate into peri-tissues, thus at least partially contributing to the therapeutic effect in SCI.

## Discussion

**Figure 6:**
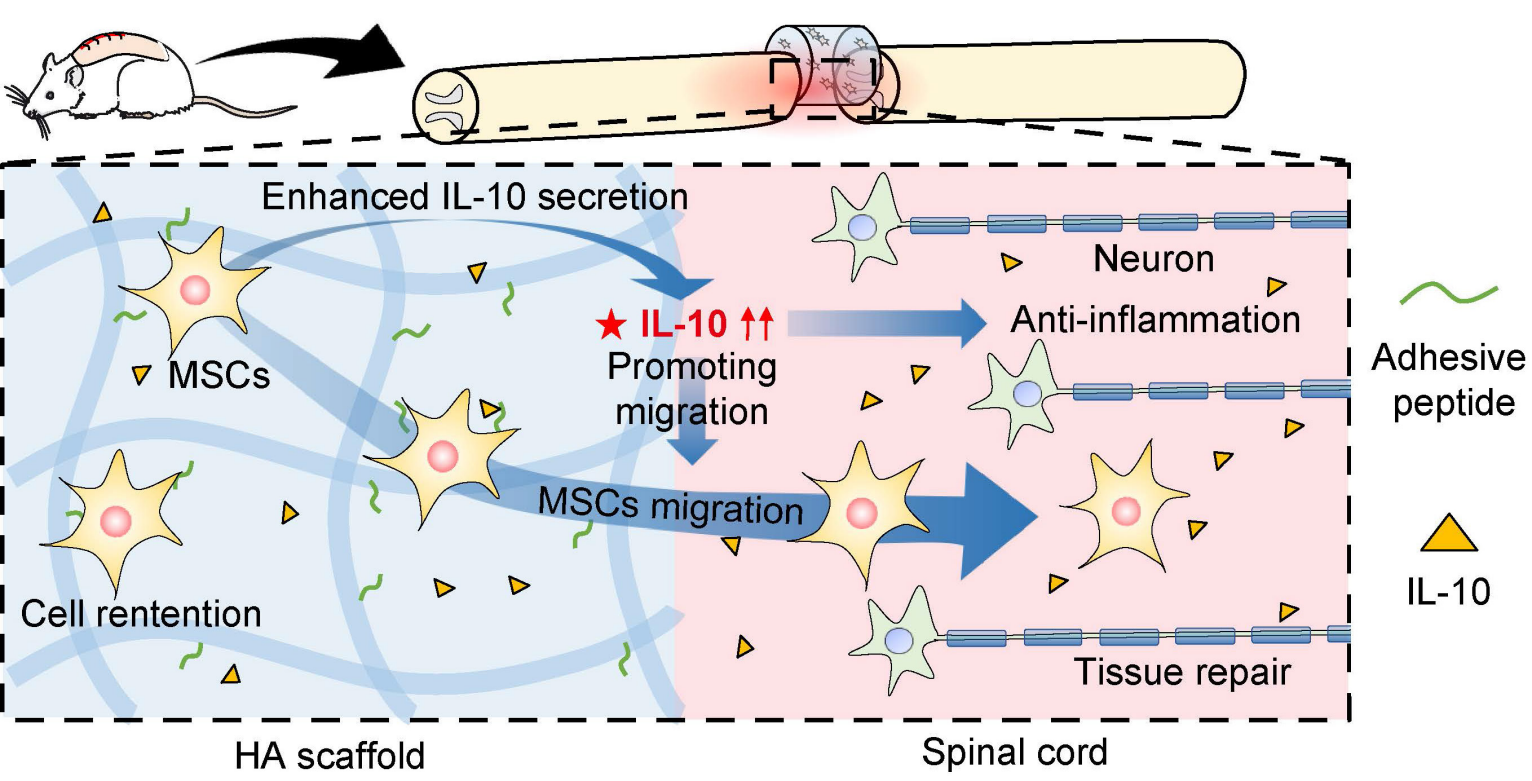
** Schematic diagram for MSC-secreted IL-10 functions in HA-MSC scaffold treatment of SCI rats.** In SCI rat model, MSCs secrete IL-10, thus promoting the cytokine secretion-associated pathway and migration of MSCs, leading to reduced inflammation and improved tissue repair in the injured spinal cord.

MSCs play a vital role in SCI treatment as they can suppress inflammation to alleviate secondary injury, differentiate into glial cells, and secrete cytokines to promote spinal cord repair 31, 32. In this study, we investigated the underlying mechanisms (Figure 6) and showed a higher retention of MSCs in SCI rats after HA-MSC scaffold transplantation, leading to the suppression of inflammation over time. Our data further highlighted that initiating human IL-10 secretion by MSCs plays a key role in SCI therapy. This was at least partially owing to IL-10 mediated increase in cytokine secretion-associated pathways and migration of MSCs.

MSC implantation has shown therapeutic effects in experimental animal models of SCI, as evidenced by functional recovery 33, 34. However, the exact function of MSCs has not been clarified so far. MSCs are directly implanted into the lesion site, and the harsh environment after SCI severely reduces their survival 35. Although many experiments have been conducted *in vitro* to culture MSCs for mechanistic studies 36, 37, exploring the mechanism of MSC therapy in SCI *in vivo* is still difficult. Our study prolonged the survival time of MSCs *in vivo* by encapsulating MSCs into HA scaffolds and found that IL-10 secreted by them plays an important role in spinal cord restoration. Thus, this study offers a new type of mechanistic research.

Additionally, MSCs have been used to repair or regenerate injured tissue in clinical tests 38. However, the low cell survival after transplantation remains a major limitation. Cell leakage or cell death caused by inflammation, oxidative stress, or other microenvironments and a lack of nutrients may limit the self-renewal of MSCs 39, 40. Hence, local accumulation of MSCs in the spinal cord is a precondition for MSC's efficacy. In the present study, we constructed an HA scaffold into which MSCs were infused. Live/dead assay showed that on day 5, there were still lots of MSCs remaining in the scaffold and some cells were observed to assemble cell sphere. MSCs secrete extracellular matrix components such as collagen 41. Hence, when MSCs gather in the cavity of the scaffold, they may gradually secrete collagen, adhere to it, and finally form cell spheres. We then implanted the scaffold into the lesion site of SCI rats and found that the cell viability increased significantly. Interestingly, we found that surviving MSCs, when attracted by inflammatory signals *in vivo* and by the action of IL-10 secreted by themselves, migrated to the vicinity of the lesion site to exert anti-inflammatory effects. These results indicate that the scaffold can prolong the residence time of MSCs *in vivo* and promote their migration to the lesion site to exert anti-inflammatory effects for better repair of SCI.

SCI causes different degrees of nerve damage, resulting in sensory and motor dysfunction and, in severe cases, quadriplegia 42. MSCs have become a research hotspot in the treatment of SCI and MSCs from different sources have been used clinically 43, 44. The regenerative capacity of MSCs is attributed to their stemness, which allows them to differentiate into glial cells 45. Moreover, recent studies have suggested that MSCs control inflammation, thereby reducing the damage to residual tissues and preventing secondary immune attacks 46. Notably, in our study, anti-inflammatory factors were significantly increased in MSC-treated rats, whereas pro-inflammatory factors were reduced, indicating the immunomodulatory effects of MSCs in SCI rats. In addition to their anti-inflammatory effects, MSCs inhibit oxidative stress and secrete extracellular vesicles 47. As expected, MDA levels decreased in the MSC-treated rats, whereas SOD levels increased. These observations suggest that MSCs function in inflammation and ROS regulation during recovery from SCI.

IL-10 is a well-known cytokine with potent anti-inflammatory and immune regulatory properties 48. Acute ischemic mice injected with IL-10 overexpressed MSCs exhibited reduced microglial activation and pro-inflammatory cytokine secretion 49. However, it is not known whether IL-10 participates in MSC treatment of SCI. Notably, MSCs secreted transient but copious amounts of IL-10, which contributed to spinal cord recovery. IL-10 did not affect MSC proliferation or apoptosis (Figure S4). However, the upregulation in cytokine secretion-associated JAK-STAT pathway was similar to previous reports, which found that IL-10 promoted MSC paracrine signaling 50. Interestingly, for the first time, we found that MSC-secreted IL-10 promoted MSC migration. IL-10 enhances the migration of tendon-derived stem cells 51; however, the migration of neutrophils is inhibited by the overexpression of IL-10 in plasmacytosis 52. Thus, the role of IL-10 in cell migration is not consistent and further studies are needed to elucidate the underlying molecular mechanisms of IL-10 regulation in MSCs. Overall, our data suggest a dual role for IL-10 in the regulation of MSC functions.

## Conclusions

In summary, we confirmed that scaffolds promoted the survival of MSCs, thereby contributing to the repression of inflammation and improving spinal cord restoration. In addition, for the first time, we highlight the important role of initiating the production of IL-10 by MSCs, which can accelerate the migration and cytokine secretion-associated pathways of MSCs, thereby contributing to the recovery of the spinal cord. These findings highlight a novel role for IL-10 in regulating MSC migration and cytokine secretion-associated pathways and identify a dominant role for IL-10 in MSC therapy for spinal cord repair.

## Supplementary Material

Supplementary figures.

Supplementary video.

## Abbreviations

SCI: spinal cord injury
MSCs: mesenchymal stem cells
HA: hyaluronic acid scaffold
IL-10: interleukin 10
HA-MSC: HA scaffold immobilized human MSCs
rhIL-10: recombined human IL-10
SOD: superoxide dismutase
MDA: malondialdehyde
ROS: reactive oxygen species
BMDM: bone-marrow-derived macrophage
PBMC: peripheral blood mononuclear cell
EMT: epithelial-mesenchymal transition
NF-κB: nuclear factor kappa B
CCR7: C-C chemokine receptor type 7
