# Supplementary material for: Initial IL-10 production dominates the therapy of mesenchymal stem cell scaffold in spinal cord injury
Source: Theranostics. 2024 Jan 1;14(2):879–91. doi: 10.7150/thno.87843 (PMC10758068; doi:10.7150/thno.87843)
Supplement: Supplementary file 1 — Supplementary figures. [file thnov14p0879s1.pdf]

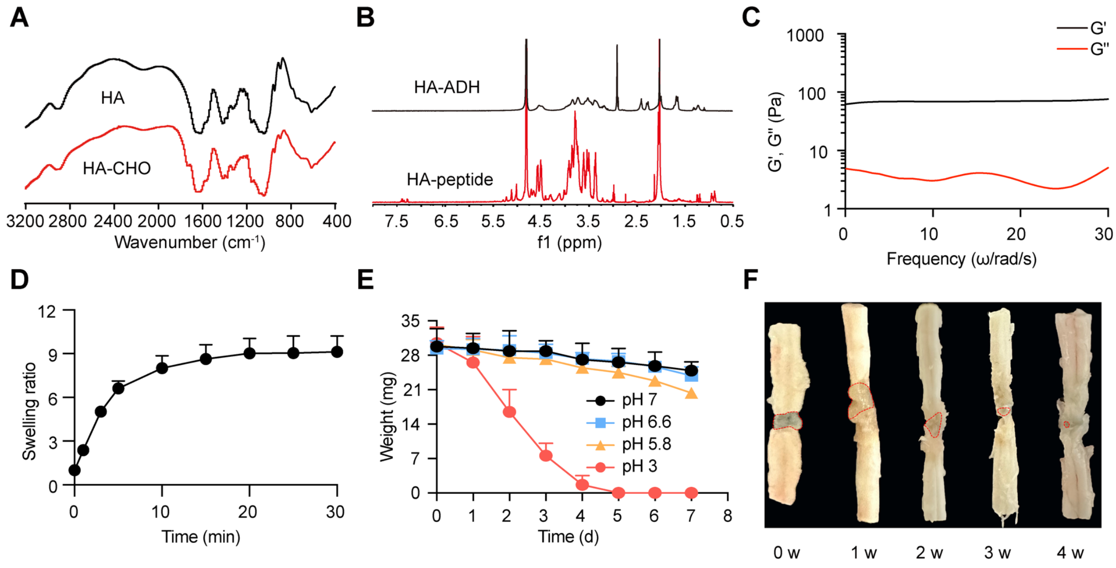


**Supplementary Figure 1 Characterization of materials for HA scaffold**

(A) FTIR spetrums of HA and HA-CHO. (B) NMR spectrums of HA-ADH and HA-peptide. (C) Rheological properties of HA scaffold. (D) Swelling behavior of HA scaffold in PBS solution (n = 3 independent experiments). (E) *In vitro* degradation of HA scaffold in PBS with different pH (7, 6.6,5.8 and 3) (n = 3 independent experiments). (F) *In vivo* degradation of HA scaffold in SCI rats. The red line indicated the scaffold.

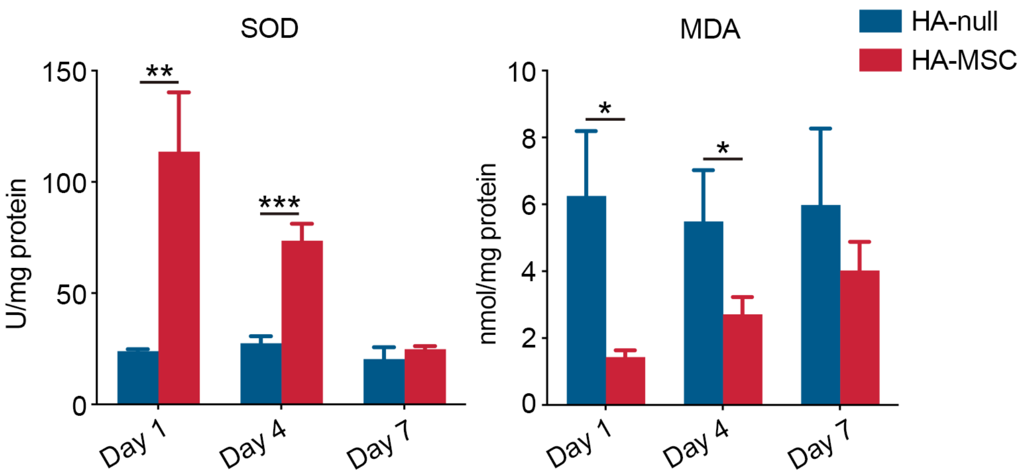


**Supplementary Figure 2 HA-MSC scaffold implantation inhibits ROS in SCI rats**

SOD and MDA levels of spinal cord in SCI rats treated with scaffold and HA-MSC on day 1, 4 and 7 (n = 3 animals per group).

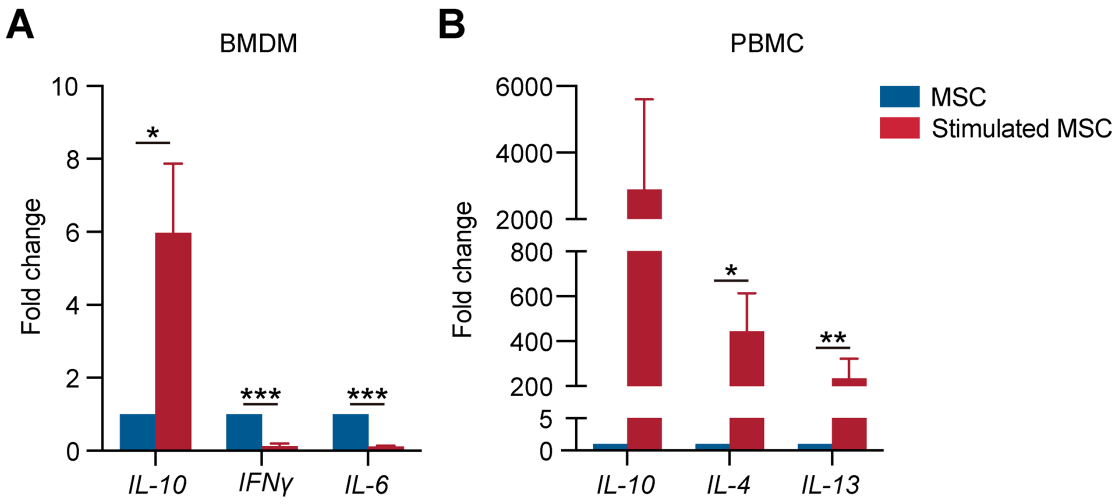


**Supplementary Figure 3 Transient increase of hIL-10 secreted by MSCs *in vitro***

(A-B) Relative mRNA expression of *IL-10* and inflammation-related factors in MSCs co-cultured with BMDM (A) or PBMC (B) for 8 h (n = 3 independent experiments).

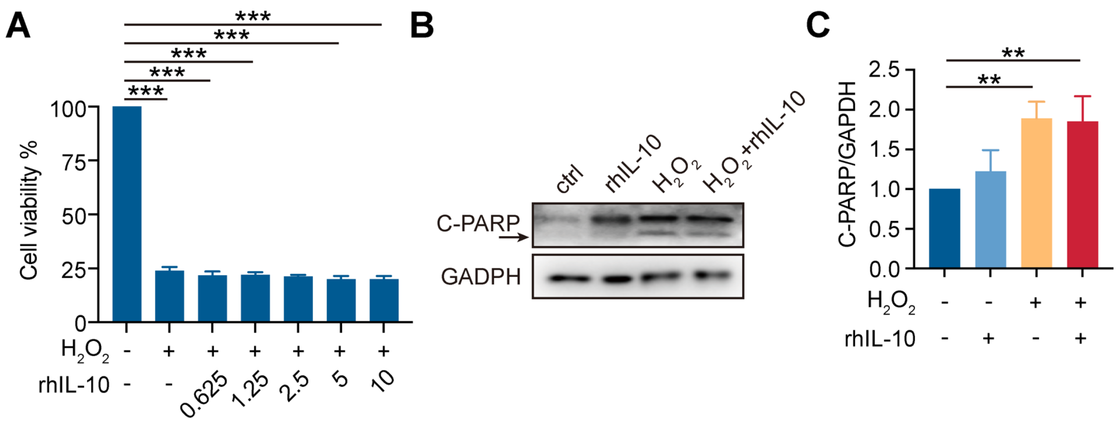


**Supplementary Figure 4 IL-10 has no effect on the proliferation and apoptosis of MSCs**

(A) Cell viability of MSCs treated with different concentrations of rhIL-10 (n = 3 independent experiments). (B-C) Western blotting analysis (B) and quantification (C) for the expression of C-PARP in the MSCs treated with rhIL-10 and $H_2O_2$ alone or together for 24 h (n = 3 independent experiments).
